# Crystalline light chain proximal tubulopathy and podocytopathy: a case report

**DOI:** 10.1590/2175-8239-JBN-2019-0086

**Published:** 2019-12-02

**Authors:** Ankit B. Patel, John Y. Choi, Walter P. Mutter, Astrid Weins, Leonardo V. Riella

**Affiliations:** 1 Harvard Medical School, Renal Division, Brigham & Women’s Hospital, Boston, MA, EUA.; 2 Harvard Medical School, Renal Division, Newton Wellesley Hospital, Boston, MA, EUA.; 3 Harvard Medical School, Brigham & Women’s Hospital, Department of Pathology, Boston, MA, EUA.

## Case presentation

In this case report we present a 64-year-old man with a history of IgG kappa paraproteinemia for 14 years with no signs of multiple myeloma or plasmacytoma who presented to the renal clinic with new onset hypokalemia and increase in serum creatinine. His past medical history was remarkable for a diagnosis of necrobiotic xanthogranuloma for 30 years treated with multiple modalities including chlorambucil, thalidomide, cryotherapy, intravenous immunoglobulin, and electron beam therapy. He also had a history of squamous cell carcinoma, melanoma, bilateral carotid artery disease, coronary artery disease, and hypertension. He had no fevers, cough, dyspnea, abdominal pain, nausea, or vomiting but did complain of a worsening headache over the last week. He also reported increased urinary frequency and thirst but denied any dysuria or hematuria. His medications included aspirin, clopidogrel, amlodipine, carvedilol, hydralazine, and atorvastatin. He started no new medications and reported no sick contacts. His blood pressure was 152/82 mmHg. On physical examination, the patient appeared comfortable with a clear oropharynx, cardiac exam with regular rate and rhythm and no murmurs, rubs, or gallops, clear lung sounds, soft and non-tender abdomen, no lower extremity edema, and numerous erythematous plaques on his trunk and extremities. His initial labs at presentation are summarized in Table 1. Of note, his creatinine was 1.0 mg/dL 10 months prior. Urine sediment examination showed occasional epithelial cells and white blood cells as well as hyaline casts. A renal ultrasound showed a right kidney of 11.9 cm with mild fullness of collecting system and a left kidney of 10.4 cm with no signs of hydronephrosis. A bone marrow biopsy 1.5 years prior to presentation showed findings consistent with a plasma cell dyscrasia but did not meet criteria for myeloma. Plasma cells were 5% without lymphoid infiltrates. Flow cytometry showed abnormal plasma cells that were kappa restricted. Cytogenetics were normal.

**Table 1:** Initial laboratory values

Variable	Reference Range	Lab value
Blood		
Sodium (mmol/L)	137-146	139
Potassium (mmol/L)	3.5-5.3	3.2
Chloride (mmol/L)	98-107	96
Carbon dioxide (mmol/L)	23-32	27
Urea nitrogen (mg/dL)	5-25	21
Creatinine (mg/dL)	0.6-1.4	1.69
Glucose (mg/dL)	70-100	120
Calcium (mg/dL)	8.6-10.3	8.9
Albumin (g/dL)	4.0-5.0	3.1
Phosphorous (mg/dL)	2.7-4.5	3.2
Magnesium (mg/dL)	1.3-2.7	1.7
Uric Acid (mg/dL)	2.6-6.0	5.6
Aspartate aminotransferase (U/L)	6-40	30
Alanine aminotransferase (U/L)	10-49	18
Alkaline phosphatase (U/L)	35-130	76
Total Bilirubin (mg/dL)	0-1.0	0.4
Hemoglobin (g/dL)	12.0-17.0	11.6
Hematocrit (%)	35.0-50.0	35.2
White-cell count (per mm^3^)	4,500-11,000	8,900
Platelet count (per mm^3^)	150,000-400,000	265,000
Kappa light chains (mg/dL)	3.3-19.4	48.7
Lambda light chains (mg/dL)	5.7-26.3	1.53
Free Kappa/lambda ratio	0.26-1.65	31.9
M-spike, SPEP (g/dL)	None	1.19 IgG Kappa
Urine		
Urinalysis		
Blood	Negative	Negative
Glucose	Negative	Negative
Protein	Negative	++
Specific gravity	1.000-1.035	1.011
pH	5.0-8.0	7.0
Sediment		
Red blood cell (/hpf)	0-3	2
White blood cell (/hpf)	0-4	1
Casts	Negative	Negative
Crystals	Negative	Negative
Chemistry		
Spot urine protein/creatinine (g/g)	< 0.15	2.94
Spot urine microalbumin/creatinine (g/g)	0-0.03	1.68
Urine potassium/Cr (mEq/g)	0-14	49.7
Urine M spike (mg/24hr)	None	244 IgG Kappa
Urine total protein (g/24hrs)	< 0.08	2.2

## Differential diagnosis

A middle age patient presented with new-onset renal dysfunction, worsening albuminuria, and increased urinary frequency and thirst. His evaluation was notable for a urinalysis with no blood but urine total protein/creatinine of 2.94 g/g and urine albumin/creatinine of 1.68 g/g. The new onset of proteinuria along with renal dysfunction helped guide the differential diagnosis for acute kidney injury. Proteinuria generally is categorized as tubular or glomerular in origin. Given the history of paraproteinemia, the new increase in proteinuria could result from an increase in the paraprotein burden. Given the modest level of free light chains and that more than half of the total protein found in the urine was albumin, the proteinuria was not due to increased urinary free light chains but rather glomerular in origin. The paraprotein can cause glomerular injury leading to albuminuria. Proteinuria can also be associated with proximal tubule dysfunction, which is considered low molecular weight proteinuria and there is a larger difference between urinary total protein and albumin excretion. Although tubular injury can lead to albuminuria, it is not typically to the degree noted in this case. The patient had a significant cancer history, although no new treatment during the time course of the renal dysfunction and no signs of recurrence of skin cancer. Of note, he did have new onset hypokalemia with urine chemistry suggestive of potassium wasting and a known underlying IgG kappa paraproteinemia.

### Hypokalemia

Hypokalemia is uncommonly associated with acute kidney injury. To determine the etiology of hypokalemia, it is important to first identify if there is a decrease in potassium intake, an increase in potassium output, or a transcellular shift of potassium. The elevated spot urinary potassium/creatinine ratio in this patient prior to potassium supplementation suggested increase urinary potassium excretion as the etiology of hypokalemia. Urinary potassium wasting can result from a number of different causes including: 1) genetic defects in tubular sodium reabsorption increasing distal sodium delivery, a key regulator of potassium excretion, 2) hypomagnesemia, which decreases tonic inhibition of secretory potassium channels, 3) varied forms of hyperaldosteronism leading to upregulation of sodium and potassium conductances in the connecting tubule and collecting duct, or 4) increase in urinary anions (e.g. bicarbonate) that are excreted with countercations such as potassium to maintain electroneutrality. Hypokalemia with acute kidney injury is a unique feature of leptospirosis, however, there was no recent travel or other clinical signs to suggest infection. Given the history of paraproteinemia and high urinary pH, proximal tubule dysfunction with proximal renal tubular acidosis (pRTA) was suspected since it may lead to bicarbonate urinary wasting and associated hypokalemia. Proximal tubulopathy from paraproteinemia is often associated with global proximal tubule dysfunction (Fanconi syndrome). However, this patient had normal serum levels of phosphorus and uric acid with no evidence of glucosuria indicating that some functions of the proximal tubule remain preserved.

### Monoclonal gammopathy with renal disease

**Figure 1:**
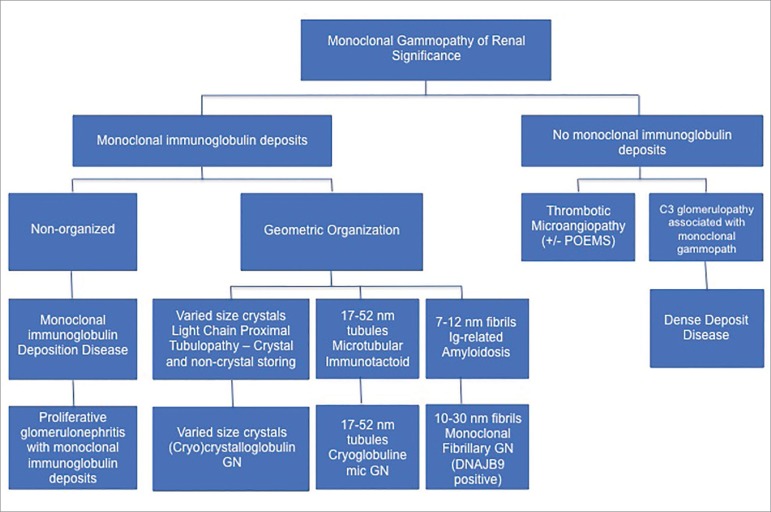
An outline of renal pathologies associated with monoclonal gammopathy of renal significance. There is an increased association of monoclonal gammopathy with disease entities denoted to have no monoclonal immunoglobulin deposits making the relation with monoclonal disease unclear. The disease entities with monoclonal immunoglobulin deposits generate organized or non-organized deposits. The organized deposits can then be differentiated based on the diameter of the individual filaments.

Monoclonal paraproteins can have an array of different effects on the kidney. Until early 2000’s, monoclonal gammopathy (MG) was categorized into monoclonal gammopathy of unknown significance (MGUS), smoldering multiple myeloma (SMM), and multiple myeloma (MM) based on: 1) monoclonal protein quantification on protein electrophoresis, 2) monoclonal plasma cell percentage on bone marrow biopsy, and 3) evidence of end organ damage such as renal failure. While this system offered some useful insight on risk stratification and prognosis, a significant proportion of MGUS population developed progressive renal disease. This observation added a new category to the previous classification: monoclonal gammopathy of renal significance (MGRS)^1^. A study conducted between January 2000 and August 2016 found 44 of 2,935 MGUS patients during that time were diagnosed with MGRS suggesting up to 1.5% of MGUS patients have renal complications attributed to their monoclonal gammopathy^2^. MGRS is further categorized based on the presence and type of organized deposits (Figure 1). If organized deposits are detected, the histopathology can be diagnosed based on the shapes and sizes on the deposits. Fibrillary deposits are usually 7-12 nm; immunotactoids are 17-52 nm hollow centered microtubules; and crystalline can present with various sizes and shapes usually in rhomboid or needle form.

### Histopathology

#### Light microscopy (LM)

**Figure 2:**
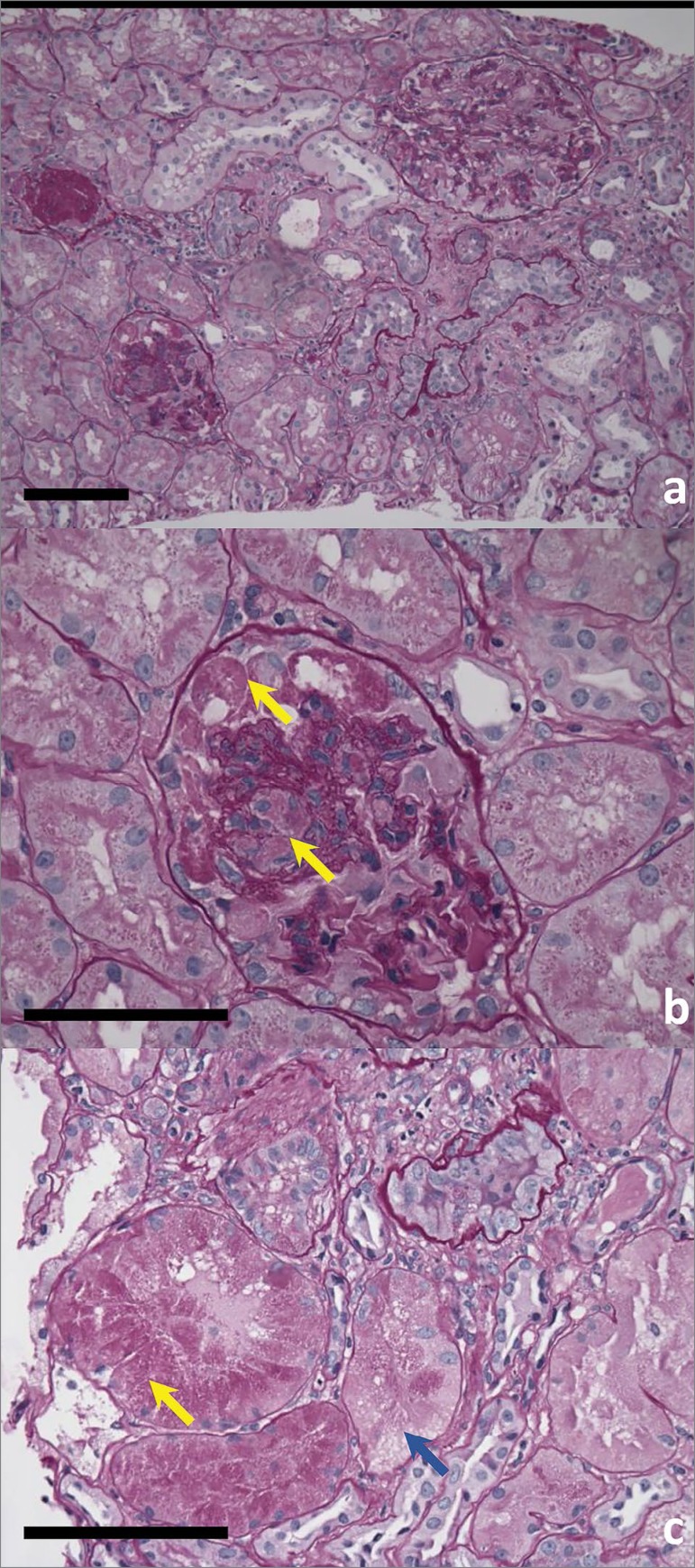
Light microscopy of kidney biopsy a) Paraffin section stained with Periodic Acid Schiff (PAS) showing three glomeruli and tubules with varying degrees of chronic changes; b) Higher magnification of a glomerulus with PAS-positive protein reabsorption granules in epithelial cells and segmental tuft collapse (arrows); c) Higher magnification of proximal tubules with PAS-positive proteinaceous granules (yellow arrow), and adjacent tubules with PAS-negative lacy cytoplasm (blue arrow). Scale bar: 50µm

A kidney biopsy was completed for further evaluation. Figure 2a shows Periodic Acid Schiff (PAS) stained kidney section with three glomeruli with varied degrees of chronic changes: one glomerulus is globally sclerosed, another glomerulus shows features of collapsing glomerulopathy, and the third glomerulus has mild mesangial expansion. There are some interspersed normal appearing tubules, some hypertrophic with obvious PAS-positive protein reabsorption granules in the cytoplasm along with basal nuclei (yellow arrow), while adjacent tubules reveal PAS-negative lacy cytoplasm (blue arrow) (Figure 2c). Figure 2b shows a higher magnification of a glomerulus with collapsing features, including extensive protein reabsorption granules in multiple epithelial cells and a collapsed tuft.

#### Immunofluorescence (IF)

**Figure 3:**
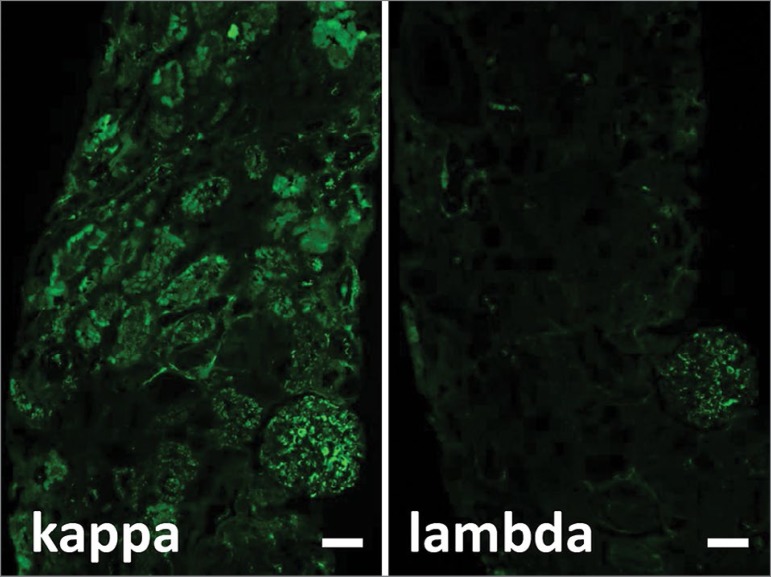
Immunofluorescence on protease-treated paraffin sections revealing kappa restricted (4+) light chain deposition in the glomerulus and tubules. Scale bar: 50µm.

Figure 3a and b shows an IF stain on paraffin sections pretreated with protease for antigen retrieval. Of note, the kappa restriction on IF can often be missed due to masking of epitopes in the ultrastructure of light chain crystals and thus paraffin sections treated with protease can help increase detection of kappa light chains by up to three-fold in patients with light chain proximal tubulopathies^3^. There was significant kappa light chain restricted staining of the proximal tubules and a mild increase in staining intensity in a single glomerulus for kappa compared to lambda light chains.

#### Electron microscopy (EM)

**Figure 4:**
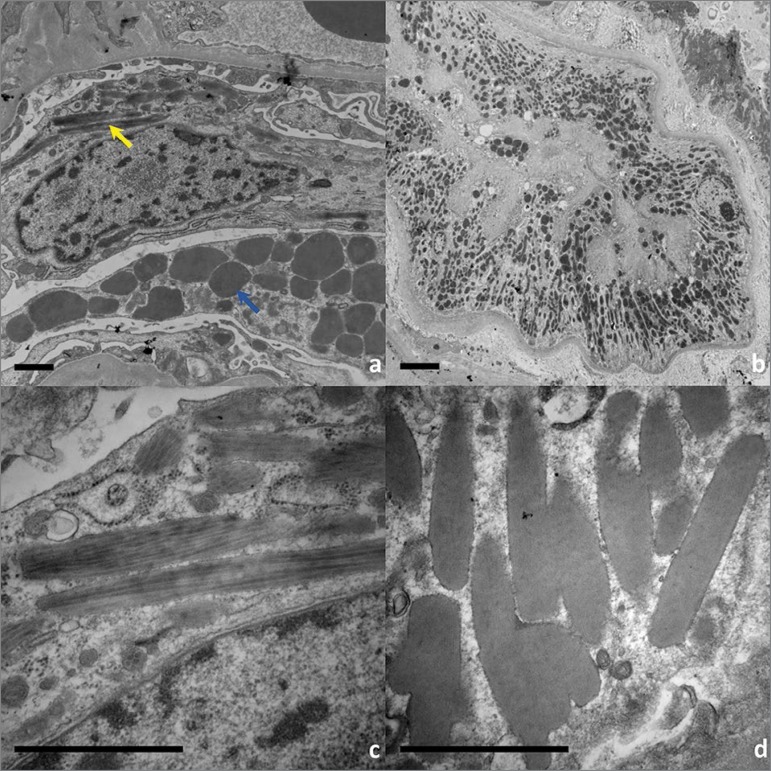
Electron microscopy demonstrating podocyte and proximal tubule crystalline deposits. a) Podocyte with foot process effacement and elongated crystal-like inclusions within lysosomes (yellow arrow) and protein reabsorption granules (blue arrow). b) Proximal tubule with numerous, distorted lysosomes and significant swelling with luminal occlusion. Higher magnification of crystals with periodic substructure found in podocytes (c) and proximal tubules (d). Scale bar: 1µm

Further evaluation with transmission electron microscopy (TEM) was completed. Figure 4a shows a podocyte with mild foot effacement but numerous needle-like crystal inclusions in lysosomes (yellow arrow) and protein reabsorption granules (blue arrow). These crystals are seen at higher magnification in 4c and show a regular, geometric substructure. The proximal tubules on TEM (Figure 4b) show significant disorganization with crystal deposition, increase in lysosomes, and complete occlusion of the lumen. Upon closer inspection, the crystals demonstrate a substructure (Figure 4d) similar to the one seen in the glomeruli.

#### Final diagnosis

The final diagnosis of the patient was crystalline light chain proximal tubulopathy and podocytopathy with collapsing features secondary to monoclonal gammopathy.

## Discussion

### Light chain proximal tubulopathy (LCPT)

LCPT is a histopathological diagnosis associated with monoclonal gammopathy that is characterized by the accumulation of light chain in the proximal tubule. The renal biopsy, in this case, showed classic features of LCPT - 1) diffuse and severe acute tubular injury with cellular hypertrophy and luminal occlusion, 2) detection of light chain (exclusively kappa restricted) in proximal tubule by immunofluorescence, and 3) intracytoplasmic crystals on electron microscopy. In the largest cohort of LCPT patients published in 2016^4^, the diagnosis was most frequently made in males with a median age of 60. Patients with LCPT often present with Fanconi Syndrome. In this case, the patient did not present with Fanconi syndrome suggesting there were enough spared proximal tubules to maintain function. There was significant albuminuria in this patient, also often not seen in LCPT patients, and shown to be glomerular in origin with evidence of collapsing glomerulopathy. Upon examination of the electron microscopy, intracytoplasmic crystals could be identified inside the podocytes. These inclusions demonstrate the ability of podocytes to endocytose proteins and light chains, a process that is conducted similarly to the proximal tubule via cubulin^5^. Though rare, a previous case report has also shown collapsing glomerulopathy associated with LCPT with similar intracytoplasmic crystals within the podocytes, summarizing the few reported cases of this association^6^. Patients with LCPT are most frequently diagnosed with MGRS followed by MM, smoldering myeloma, non-Hodgkin lymphoma, and chronic lymphocytic leukemia. During a median follow-up period of 39 months, patients treated with stem cell transplantation and chemotherapy showed lower mortality and renal failure when compared to an observation group suggesting the value of aggressive intervention. However, authors also pointed out that patients who did not receive stem cell transplantation nor chemotherapy were older and had higher initial serum creatinine.

### Pathophysiology of light chain intracellular aggregation

Intracellular immunoglobulin light chain aggregation is shown to be associated with biochemical properties of individual light chain clones. A review of patients with light chain proximal tubulopathy showed that the characteristics of the pathogenic urinary light chain is quite homogenous despite the phenotypic heterogeneity in patients and their clinical course. Of the 9 patients with urinary light chains analyzed, 8 had V kappa I variability subgroup with similar characteristic residues at key positions^7^. A key feature of the V kappa I variability subgroup was its resistance to proteolysis. A separate study evaluated the protease resistance of a specific 12 kDa fragment of the light chain in patients with cast nephropathy or LCPT by cathepsin B. In this study, there was complete or partial degradation of the light chains from 12 cast nephropathy patients and 4 control patients while 4 patients with LCPT had no degradation of 12 kDa fragment by cathepsin B^8^. This data suggests that proteolysis-resistant light chains are associated with LCPT and due to impaired degradation overwhelm the proximal tubule lysozymes ultimately leading to light chain aggregation and tubular dysfunction.

### Follow-Up

The patient was referred to the hematology/oncology clinic where a bone marrow core biopsy showed a normocellular bone marrow with less than 5% plasma cells suggestive of plasma cell dyscrasia without overt multiple myeloma. He was treated with cyclophosphamide, bortezomib, and dexamethasone. He briefly had stabilization of kidney function with decrease in paraprotein but developed recurrent disease 1.5 years later. A repeat bone marrow biopsy at that time showed 15% plasma cells with kappa light chain dominance consistent with overt myeloma. He was then started on treatment with daratumumab and bortezomib. A repeat bone marrow biopsy showed marked hypocellularity and less than 5% plasma cells, however, his kappa light chain production continued to be high. He ultimately had progressive chronic kidney disease and was started on hemodialysis three years after presentation.
